# Comparison of the efficacy and adverse effects of unilateral or bilateral botulinum toxin injections for adductor spasmodic dysphonia: a systematic review and meta-analysis

**DOI:** 10.1007/s00405-023-08366-2

**Published:** 2023-12-14

**Authors:** Yuyin Liu, Feifei Chen, Fangqi Liang, Can Wang, Dan Chen, Jing Zhou, Lu Zhang, Xiao Xiao, Ronghua He, Li Tang, Li Tian, Li Zhou

**Affiliations:** Department of Otorhinolaryngology, Hospital of Chengdu University of Traditional Chinese Medicine, No.39 Shi-Er-Qiao Road, Chengdu, 610072 Sichuan Province People’s Republic of China

**Keywords:** Spasmodic dysphonia, Adductor, Botulinum toxin, Meta-analysis, Systematic review

## Abstract

**Purpose:**

This study aims to aggregate and analyze existing clinical evidence to compare the efficacy and adverse effects of unilateral or bilateral botulinum toxin injections for the treatment of adductor spasmodic dysphonia (ADSD).

**Methods:**

Reports from non-randomized controlled trials and cohort studies pertaining to the efficacy and adverse effects of unilateral and bilateral botulinum toxin injections for ADSD were identified and retrieved from four electronic databases from inception to July 2023. The meta-analysis employed fixed or random effects models to assess pooled relative risks (RR), mean differences (MDs), and standard mean differences (SMDs) with their corresponding 95% confidence intervals (CIs).

**Results:**

We included two non-randomized controlled trials and seven cohort studies comprising 854 total patients. Meta-analysis of the included studies showed that bilateral botulinum toxin injections associated with a longer duration of vocal improvement (MD =  − 2.89, 95% CI − 3.13 to − 2.65, *I*^2^ = 0%, *P* < 0.00001). However, bilateral botulinum toxin injections associated with an increase in adverse effects, including a longer duration of breathy voice quality (SMD =  − 0.51, 95% CI − 0.79 to − 0.22, *I*^2^ = 35%, *P* = 0.0005) and a higher occurrence of swallowing difficulties (RR = 0.46, 95% CI 0.35 to 0.11, *I*^2^ = 0%, *P* < 0.00001).

**Conclusion:**

Bilateral botulinum toxin injections for ADSD showed a longer duration of vocal improvement, a longer breathy voice duration and a higher dysphagia occurrence and duration than unilateral injections.

## Introduction

Spasmodic dysphonia (SD), also known as laryngeal dystonia (LD), is a focal dystonia primarily characterized by irregular and uncontrollable interruptions in phonation that are commonly accompanied by vocal effort [1, 2]. The reported prevalence of SD varies between 0.9 and 13.7 per 100,000 individuals worldwide [3]. Due to diagnostic challenges and the lack of a global epidemiological investigation, the true prevalence remains underestimated.

SD can be further classified based on the affected muscle groups as either adductor spasmodic dysphonia (ADSD), abductor spasmodic dysphonia (ABSD), or mixed-type dysphonia, with ADSD being the most common subtype (accounting for 90–95% of cases) [4]. The irregular voice breaks caused by laryngeal muscle spasms have been shown to significantly impair patient quality of life, leading to decreased work attendance and performance [5, 6]. Furthermore, anxiety and depression coexist in 7.1–62% of ADSD patients [7], and approximately one-fifth of ADSD patients experience suicidal ideation [8].

Injecting botulinum toxin into the affected muscles for treating SD has a history of over 40 years. Blitzer et al. [9] demonstrated the effectiveness of local botulinum toxin injection for ADSD in 1986. The 2018 update to the Clinical Practice Guideline: Hoarseness (Dysphonia) [10] explicitly recommended botulinum toxin injection as a treatment for patients with voice disorders caused by SD. It is estimated that nearly 85% of SD patients are treated with botulinum toxin injections in modern clinical practice [11].

Following botulinum toxin injection, SD patients usually experience three stages of vocal improvement: first, a “weak breathy voice,” progression to a “normal strong voice,” and finally reversion to “spasmodic phonation” [12] during which the therapeutic effects of botulinum toxin often coexist with adverse effects. Previous studies have demonstrated that duration of vocal improvement after botulinum toxin injection typically ranges from 8.0 to 15.1 weeks, and is accompanied by a variety of adverse effects which may be influenced by individual differences (e.g., breathy voice, dysphagia/aspiration, coughing, and complete loss of voice) [13–15]. Clinicians often tailor personalized treatment plans based on the severity of the vocal disorder, diverse patient needs for voice recovery, botulinum toxin treatment efficacy, and patient feedback on adverse effects.

Therefore, a key question remains: is there a way to optimize treatment efficacy and minimize adverse effects for SD at the lowest economic cost? Numerous studies have explored different injection approaches, including comparing the efficacy and adverse effects of unilateral and bilateral botulinum toxin injections for ADSD. However, due to discrepancies in study design, outcome measures, and research findings, there is currently no unified consensus on whether unilateral or bilateral botulinum toxin injection is superior in terms of efficacy and adverse effect profile. Thus, the present study aims to aggregate and analyze existing evidence regarding the efficacy and adverse effects of unilateral and bilateral botulinum toxin injections for ADSD. These findings may provide insight needed to optimize the clinical approach to botulinum toxin injection treatment for ADSD.

## Methods

This study adhered to the PRISMA 2020 recommendations [16] and employed a pre-established protocol and a clear, replicable literature search strategy for the systematic review and meta-analysis of existing evidence. The study has been registered with PROSPERO (Registration Number: CRD42023451772).

### Study selection

The inclusion criteria were formulated according to the Population, Intervention, Comparison, Outcomes and Study (PICOS) principle:

1. Population: adult patients diagnosed with ADSD (with or without tremor).
2. Intervention: unilateral botulinum toxin injections (regardless of side).
3. Comparison: bilateral botulinum toxin injections.
4. Outcomes: both efficacy evaluation and adverse effect assessment were considered. Efficacy evaluation included the duration of vocal improvement, injection interval, and relevant severity scores for vocal disorders. Adverse effect assessment encompassed the duration or occurrence rate of symptoms such as complete loss of voice, breathy voice, swallowing difficulties, and coughing.
5. Study: non-randomized controlled trials and cohort studies.

Exclusion criteria consisted of duplicates, unclear study design, articles without access to original data or full texts, animal studies, case reports, reviews, and systematic reviews.

### Search and screening strategy

The search was conducted using four medical electronic databases (PubMed, Embase, Web of Science, and Cochrane Library), scanning for all articles published up to July 2023. Language, date, and geographical restrictions were not applied in the literature search. A combination of subject terms and free-text terms were used to construct the search; the complete search strategy is provided in the appendix. References included within relevant articles were additionally scrutinized, and experts in the field were contacted to confirm any ongoing but unpublished relevant research.

The literature screening process involved the following steps: initially, two reviewers (LYY and CD) independently screened the titles and abstracts of relevant literature from each major electronic database. The results of their screenings were then compared. Studies that overlapped (i.e., articles identified as relevant by both reviewers) proceeded to the next screening step directly. For non-overlapping studies, a discussion was held to reach a consensus on whether or not they should transition to the next screening step. If discrepancies persisted, a third team member (ZJ) was consulted to reach a consensus. Subsequently, a comprehensive full-text screening of the preliminarily screened literature was conducted. LYY and CD independently read the full texts and determined inclusion or exclusion based on pre-defined criteria. The final inclusion of articles was determined through a process of comparison and discussion, as outlined previously.

### Data extraction and critical appraisal

LYY and CD independently used a standardized data extraction template to isolate relevant information and data from the included literature. The data extracted by the two reviewers were examined and compared by an independent third party. In cases of discordant data extraction, consensus was achieved through discussion. A quality assessment of the bias present in the included studies was conducted using the ROBINS-I tool [17], which categorizes literature quality into one of five levels: low risk of bias, moderate risk of bias, high risk of bias, critical risk of bias, or no information.

### Statistical analysis

A meta-analysis was subsequently performed on the collated data derived from the included literature. Descriptive summaries were provided for studies with sample sizes > 10. For studies with two or more research papers focusing on a specific outcome, Review Manager software (version 5.4) was used to aggregate effect sizes. Continuous variables were reported using mean differences (MDs) or standardized mean differences (SMDs) along with the associated 95% confidence interval (CI). Binary variables were reported using relative risk (RR) and the associated 95% CI. Heterogeneity was evaluated using the I-squared (*I*^2^) statistic, which describes the degree of between-study variability. If the heterogeneity was low (*I*^2^ < 50%), the fixed-effect model was adopted for analysis. If heterogeneity was high (*I*^2^ > 50%), we performed a sensitivity analysis to observe the study with the greatest heterogeneity and excluded this study from the analysis. Additionally, we conducted a subgroup analysis according to the actual situation of the study to reduce heterogeneity.

## Results

**Fig. 1:**
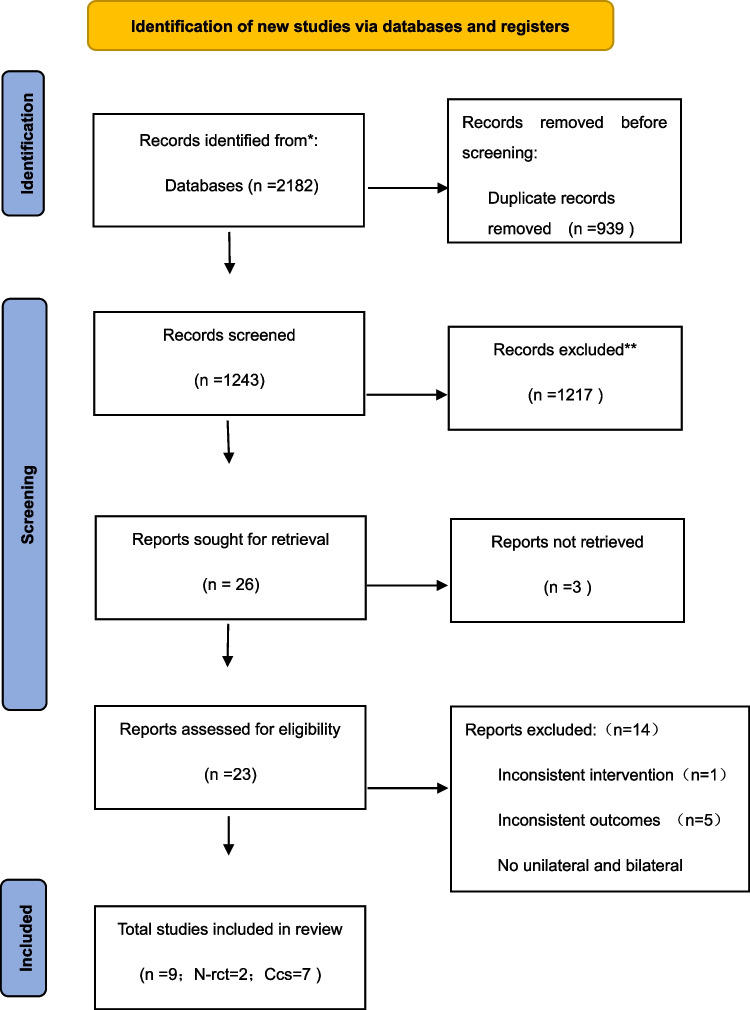
PRISMA flow diagram. *PubMed = 408; Cochrane = 44; Web of Science = 215; Embase = 1515. **Abstracts and titles screened for relevance (*n* = 1185); conference papers, reports, and review articles (*n* = 30). Others (protocols, letters, technical notes, animal studies, non-English language; *n* = 2). N-rct, non-randomized controlled trials; Ccs, cohort studies. From: Page MJ, McKenzie JE, Bossuyt PM, Boutron I, Hoffmann TC, Mulrow CD, et al. The PRISMA 2020 statement: an updated guideline for reporting systematic reviews. BMJ 2021;372:n71. https://doi.org/10.1136/bmj.n71

A total of 2182 records were retrieved from various electronic databases as follows: PubMed = 408; Cochrane Library = 44; Web of Science = 215; Embase = 1515. After excluding 939 duplicate records and reviewing titles and abstracts, a final set of 9 articles were included for this study. These articles consisted of seven cohort studies and two non-randomized controlled trials, with a collective sample size of *n* = 854 cases. The study selection process is detailed in a PRISMA diagram (Fig. 1).

### Study characteristics and risk-of-bias assessment

The main characteristics of the literature included in this study are shown in Table 1.

**Table 1:** Characteristics of included studies

Study reference	Study design	Study period	Patients included	Sample size	Injecting schedule	Botox manufacturer	Injecting dose (units)	Efficacy endpoints	Adverse effect endpoints
1. Amy et al. USA [18]	Retrospective cohort study	1990–2021	ADSD (+ ETV)	(1) (alternating) *n* = 223 (2) (equal dose) *n* = 45	EMG guided Injection inserted through the cricothyroid membrane into the TA muscle	Botox	(Per site) (1) and (2) = 0.5–7.5	A (week)	D (week); E’
2. Seung et al. USA [19]	Retrospective cohort study	2005–2017	ADSD	(1) (alternating) *n* = 105 (2) = 32	EMG guided Injection inserted through the cricothyroid membrane into the TA muscle	Botox	(Per site mean does) (1) = 1.548 ± 0.434 (2) = 1.205 ± 0.317	B (month)	D (week)
3. Gabrielle et al. Canada [20]	Retrospective cohort study	1996–2017	ADSD	(1) *n* = 17 (2) *n* = 107	EMG guided Injection inserted through the cricothyroid membrane into the TA muscle	Botox	(Per site mean does) (1) = 1.65 ± 0.62 (2) = 1.28 ± 0.41	B (day)	–
4. Behrad et al. UK [21]	Retrospective cohort study	2000–2007	ADSD	(1) *n* = 12 (2) *n* = 19	EMG guided Injection inserted through the cricothyroid membrane into the TA muscle	Dysport	(Total mean does) (1) = 3.5 ± 0.02 (2) = 3.25 ± 0.03	B (day); C	E (day); F (day); G (day)
5. Upile et al. UK [22]	Retrospective cohort study	1998–2006	ADSD	(1) *n* = 11 (2) *n* = 20	EMG guided Injection inserted through the cricothyroid membrane into the TA muscle	Dysport	(Total mean does) (1) = 3.6 ± 0.02 (2) = 6.6 ± 0.02	B (day); C	D (day); E (day); E’; F; G

Study reference	Study design	Study period	Patients included	Sample size	Injecting schedule	Botox manufacturer	Injecting dose (units)	Efficacy endpoints	Adverse effect endpoints
6. Langeveld et al. [15] Netherlands	Non-randomized control	1998	ADSD	(1) *n* = 20 (2) *n* = 20	EMG guided Injection inserted through the cricothyroid membrane into the TA muscle	Botox	(Total does) (1) = 5 (2) = 2.5/per site	A (week)	D (day); E (day); E’
7. Michael et al. USA [12]	Retrospective cohort study	1989–1994	ADSD	(1) (alternating) *n* = 18 (2) *n* = 18	EMG guided Injection inserted through the cricothyroid membrane into the TA muscle	Botox	(The initial dose) (1) = 2 (2) = 2.5	B (day)	E (day)
8. Katsuhide et al. USA [23]	Retrospective cohort study	1988–1995	ADSD	(1) *n* = 57 (2) *n* = 106	Indirect laryngoscopic guided Injection inserted into the TA muscle	Botox	(per site mean does) (1) = 3.97 ± 1.06 (2) = 4.72 ± 0.83	A (week); B (day)	–
9. Zwirner et al. USA [24]	Non-randomized control	1993	ADSD	(1) *n* = 11 (2) *n* = 13	EMG guided Injection inserted through the cricothyroid membrane into the TA muscle	Botox	(dosage range) (1) = 5–30 (2) = 1.5–2.5	–	D (day); E’

*ETV* essential tremor of the voice, (1) unilateral injection regimen, (2) bilateral injection regimen, *A *length of improved voice, *B* treatment interval, *C* Patient self-assessment of Voice Score, *D* Duration of breath voice, *E* Dysphagia duration, *E’* Dysphagia occurrence, *F* Duration of coughing, *G* Duration of total voice los

The quality and bias risk assessments for the nine included studies were conducted using the ROBINS-I tool. First, a reference target randomized controlled trial (RCT) was established with the following specific parameters: the trial type was RCT, the study population consisted of ADSD patients, the intervention in the experimental group received unilateral botulinum toxin injections, and the control group received bilateral botulinum toxin injections. Outcome measures were categorized based on the design of each individual study, which could include both intention-to-treat analysis results and as-treated analysis results. These outcome measures were defined as the duration of vocal improvement, injection interval, duration of breathy voice, duration and frequency of swallowing difficulties, duration of coughing, and duration of complete voice loss.

Following this, the identification of potential confounding factors was facilitated through literature review and integration of specialized knowledge. The severity of vocal disorders in ADSD patients was identified as a crucial confounding domain, as it could influence the decision-making process of patients and physicians when choosing between unilateral or bilateral botulinum toxin injections for treatment. Additionally, consideration was given to the presence of any external interventions (e.g., speech training), which could have affected the studies.

Ultimately, by addressing key questions, the risk of bias was assessed for each study across different domains, leading to an overall evaluation of bias-associated risk after aggregation (Table 2).

**Table 2:** Risk of bias in the studies evaluated using the ROBINS-1 tool

References	C	S^*^	I	D	M	O^*^	S^**^	O^**^
Amy et al. [18]	**	**	**	**	*	**	*	**
Lee et al. [19]	***	*	**	*	*	**	*	***
Gabrielle et al. [20]	***	**	**	**	*	**	*	***
Behrad et al. [21]	***	*	**	*	*	**	*	***
Upile et al. [22]	***	*	*	*	*	**	**	***
Langeveld et al. [15]	*	*	*	*	*	**	**	**
Michael et al. [12]	***	**	**	**	*	**	**	***
Katsuhide et al. [23]	***	*	**	*	*	**	*	***
Zwirner et al. [24]	*	*	*	*	*	**	**	**

*C* Confounding factors, S^*^ Selection bias, *I* Intended interventions, *D* Deviations from interventions, *M* Missing data, *O* Outcome measurements, S^**^ Selection of reported results, O^**^ Over all bias

*Low risk of bias

**Moderate risk of bias

***Serious risk of bias

****Critical risk of bias

*****No information

## Efficacy

### Duration of vocal improvement

**Fig. 2:**
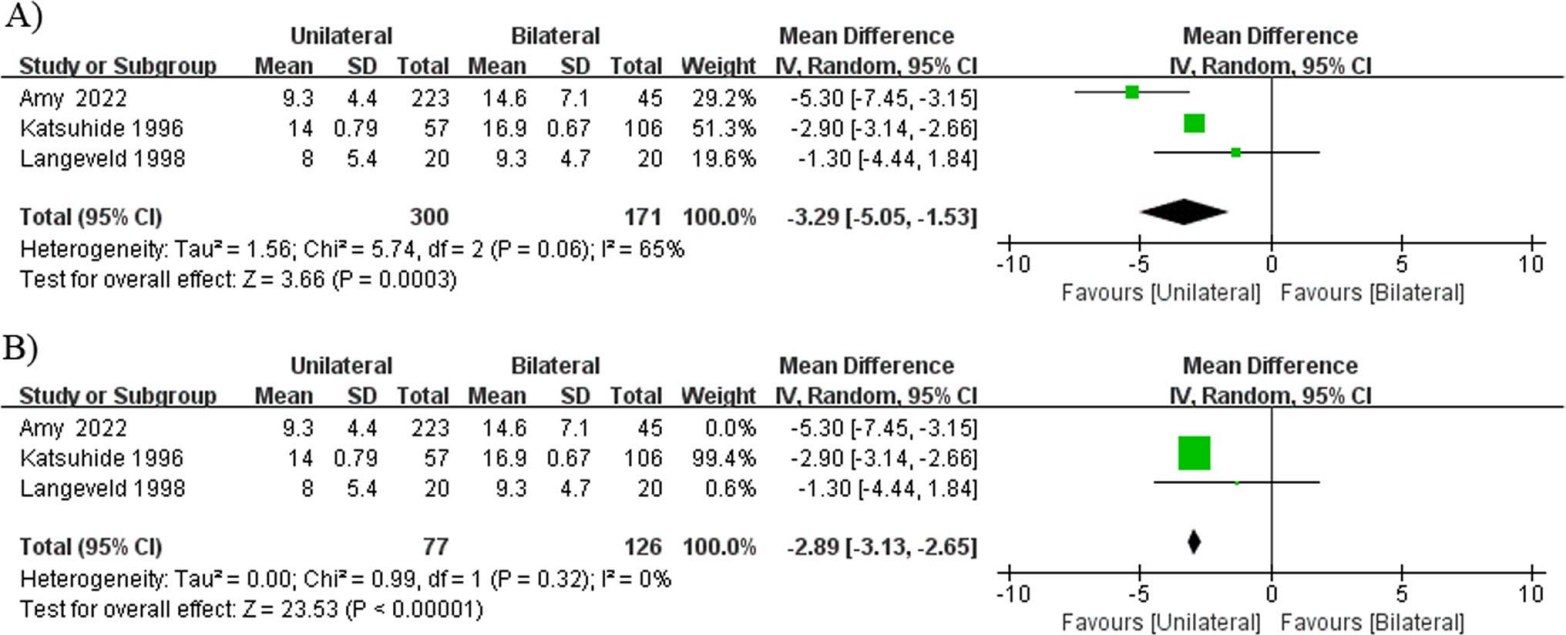
Forest plot for the meta-analysis of duration of improved voice

Results regarding the duration of improved voice were reported in two retrospective cohort studies [18, 23] and one non-randomized controlled study [15].The mixed duration of improved voice demonstrated significant difference, but showed significant heterogeneity (*I*^2^ = 65%, *P* = 0.06), which was completely reduced by excluding the study by Amy et al. from the analysis [18] (*I*^2^ = 0%, *P* = 0.32), and the result remained that unilateral injections resulted in a significantly shorter duration of improved voice compared with bilateral injections (MD =  − 2.89, 95% CI − 3.13 to − 2.65, *P* < 0.00001; Fig. 2) [15, 23].

### Treatment interval

**Fig. 3:**
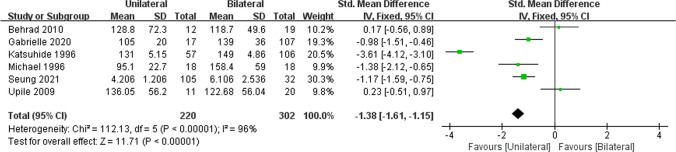
Forest plot for the meta-analysis of injection interval

The results pertaining to the treatment interval were reported in five retrospective cohort studies [12, 19–21, 23] and one prospective cohort study [22]. Among these, one study [12] did not explicitly provide averages (i.e., mean ± SD) for injection interval times in each group. Regardless, a meta-analysis was not performed upon these data due to evidence of high heterogeneity (*I*^2^ = 96%, *P* < 0.00001; Fig. 3), and we could not reduce the heterogeneity using the leave-one-out statistical method.

### Patient self-assessment of voice score

Two studies, conducted by Upile [22] and Behrad [21], explored the injection interval and also quantified the degree of vocal improvement. Patients in these studies provided a self-assessment on the extent of vocal improvement following botulinum toxin injections using the following six-point scale: 0—no improvement; 1—very slight improvement; 2—slight improvement; 3—moderate improvement; 4—marked improvement; 5—extreme improvement/near normal. Upile’s findings [22] demonstrated that the unilateral injection group had a higher vocal score (4.24) compared to the bilateral injection group (3.93). Similarly, Behrad [21] found that the proportion of patients in the unilateral injection group achieving a vocal score of 5 (near normal) was higher (54.2%) than in the bilateral injection group (46.1%).

### Adverse effects

#### Duration of breathy voice

**Fig. 4:**
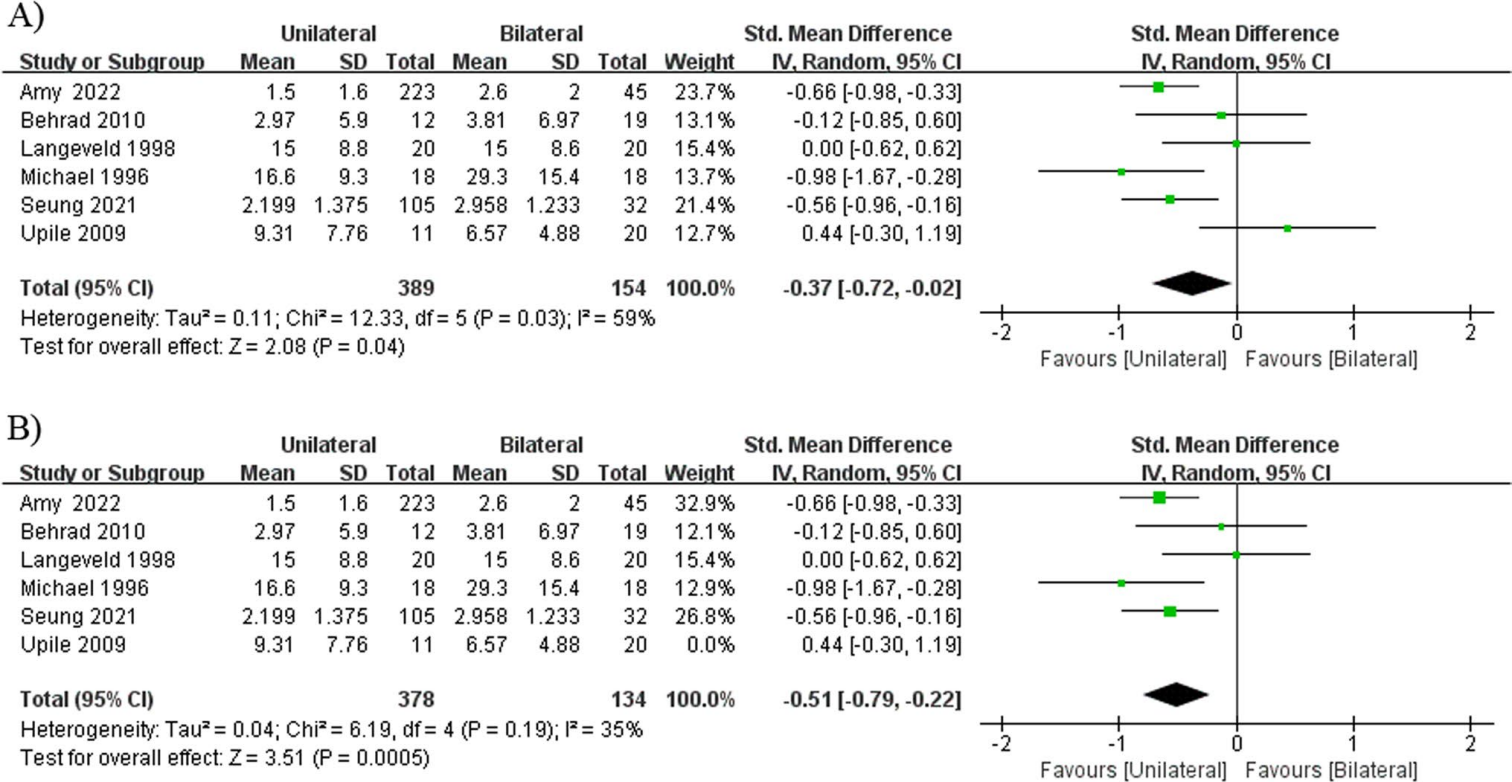
Forest plot for the meta-analysis of duration of breathy voice

The length of time that patients experienced a breathy voice quality was reported in four retrospective cohort studies [12, 18–20], one prospective cohort study [22], and one non-randomized controlled study [15].The mixed duration of breathy voice demonstrated significant difference, but showed significant heterogeneity (*I*^2^ = 59%, *P* = 0.03), which was completely reduced by excluding the study by Upile et al. from the analysis [22] (*I*^2^ = 35%, *P* = 0.19), and the result remained that unilateral injections resulted in a significantly shorter duration of breathy voice compared with bilateral injections (SMD =  − 0.51, 95% CI − 0.79 to − 0.22, *P* = 0.0005; Fig. 4) [12, 15, 18–20].

#### Dysphagia occurrence and duration

**Fig. 5:**
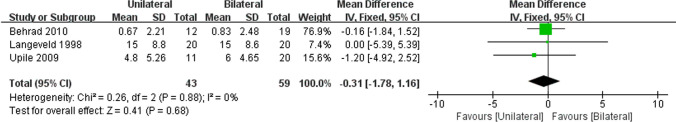
Forest plot for the meta-analysis of dysphagia duration

The occurrence and duration of dysphagia were reported in one retrospective cohort study [21], one prospective cohort study [22], and one non-randomized controlled study [15]. No significant difference in the duration of dysphagia was found between the two groups (SMD =  − 0.31, 95% CI − 1.78 to 1.16, *P* = 0.68, *I*^2^ = 0; Fig. 5).

**Fig. 6:**
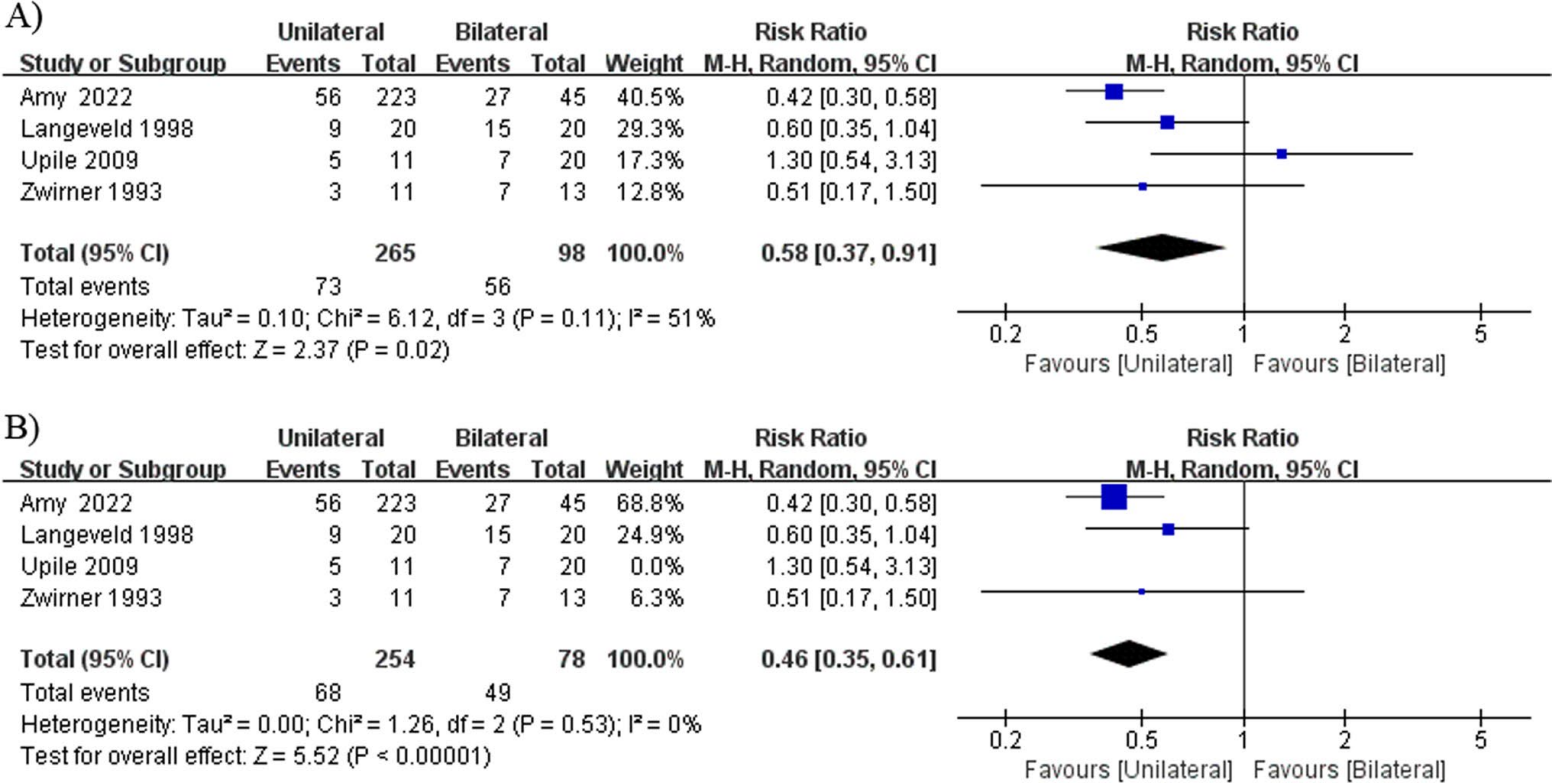
Forest plot for the meta-analysis of dysphagia incidence

The rate of occurrence of dysphagia was reported in one retrospective cohort study [18], one prospective cohort study [22], and two non-randomized controlled studies [15, 24]. The mixed rate of occurrence of dysphagia demonstrated significant difference, but showed significant heterogeneity (*I*^2^ = 51%, *P* = 0.11), which was completely reduced by excluding the study by Upile et al. from the analysis [22] (*I*^2^ = 0%, *P* = 0.53), and the result remained that unilateral injections resulted in a significantly lower rate of occurrence of dysphagia compared with bilateral injections (RR = 0.46, 95% CI 0.35 to 0.61, *P* < 0.00001; Fig. 6) [15, 18, 24].

#### Duration of coughing

**Fig. 7:**
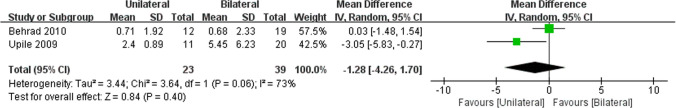
Forest plot for the meta-analysis of duration of coughing

The duration of coughing following treatment was reported in one retrospective cohort study [21] and one prospective cohort study [22].The pooled mean difference showed no significant difference (MD =  − 1.28, 95% CI − 4.26 to 1.70, *P* = 0.40; Fig. 7) in the duration of coughing. However, the result was significantly heterogeneous (*I*^2^ = 73%, *P* = 0.06), and we could not reduce the heterogeneity using the leave-one-out statistical method.

#### Duration of total voice loss

The duration of total voice loss was reported in one retrospective cohort study [21] and one prospective cohort study [22]. However, due to the absence of cases with complete voice loss in the unilateral injection group in one study [22], it was not possible to pool the results. Nevertheless, the evident trend in both studies associates a longer duration of complete voice loss with bilateral botulinum toxin injections.

The collective study findings presented above, along with the GRADE evaluation, are summarized in Table 3.

**Table 3:** Summary of study findings and GRADE evaluation

Outcomes	Number of participants (studies)	Certainty of the evidence (GRADE)	Relative effect (95% CI)
Efficacy			
Duration of improved voice	471 (2Ccs + 1N-rct)	⊕ ⊕ ⊝ ⊝ LOW	MD − 2.89 (− 3.13 to − 2.65)
Treatment interval	432 (6Ccs)	⊕ ⊝ ⊝ ⊝ VERY LOW^a^	–
Adverse effect			
Duration of breathy voice	543 (5Ccs + 1N-rct)	⊕ ⊕ ⊝ ⊝ LOW	SMD − 0.51 (− 0.79 to − 0.22)
Duration of dysphagia	102 (2Ccs + 1N-rct)	⊕ ⊕ ⊝ ⊝ LOW	SMD − 0.31 (− 1.78 to 1.16)
	363 (2Ccs + 2N-rcts)	⊕ ⊕ ⊝ ⊝ LOW	RR 0.46 (0.35 to 0.61)
Duration of coughing	62 (2Ccs)	⊕ ⊕ ⊝ ⊝ LOW	–
Duration of total voice loss	62 (2Ccs)	⊕ ⊕ ⊝ ⊝ LOW	–

*N-rct* non-randomized controlled trials, *Ccs* cohort studies

^a^Downgraded once for inconsistency (heterogeneity) in the results

## Discussion

The objective of this study was to compare the efficacy and adverse effects of unilateral and bilateral botulinum toxin injections for treating ADSD. The systematic review was based upon evidence derived from seven cohort studies and two non-randomized controlled trials. The results were pooled and evaluated through meta-analysis to gain insight into the effect of laterality on overall treatment efficacy and side effect profile.

The efficacy outcome measures in the studies investigating single versus bilateral injections included both subjective patient self-assessments (e.g., duration of voice improvement, patient-determined injection interval, maximum efficacy, and voice score), as well as objective assessments [24] made based upon acoustic analyses. However, since ADSD has a profound effect on patient experience and quality of life, most studies used subjective assessments as the primary outcome measures.

A previous meta-analysis that is comparable to the present report [25] pooled data from 22 studies, using pre- and post-treatment efficacy of different injection approaches (unilateral/bilateral) as the primary outcome. Data derived from 134 patients were evaluated, and no significant difference was identified between the two injection approaches. In this report, evidence was obtained from seven cohort studies and two non-randomized controlled trials, representing data from a total of 854 patients. The duration of voice improvement post injection and the injection interval were used as outcome measures to assess the impact of injection approach on efficacy.

After sensitivity analysis was used to exclude articles with the greatest heterogeneity, the pooled results showed that bilateral Botox injection was associated with a longer duration of voice improvement than unilateral injection. In terms of injection interval, the high heterogeneity among various studies could not be reduced, which was considered to be due to the fact that none of the included studies placed timing restrictions on retreatment schedules following a failure of patient improvement, leading to significant bias from variability in patient circumstance. Therefore, data could not be pooled or analyzed for this outcome measure. To address this issue, Steven et al. [26] categorized the reinjection interval after symptom recurrence as either “ < 2 weeks” or “ > 2 months” and compared the injection interval of unilateral versus bilateral injections. They found no significant difference between unilateral and bilateral injections for patients reinjected at < 2 weeks or > 2 months after recurrence.

Notably, the severity of voice impairment in patients with ADSD significantly impacts the treatment efficacy, side effects, risk of bias, and quality assessment reported by studies. Nonetheless, only two of the studies included in this report [21, 22] considered the severity of voice impairment in the evaluation of treatment efficacy, and these reported findings using a simple and subjective six-point rating scale. There is a significant need for more scientific, objective, and ADSD-specific measures of disease severity. Since the data types of the final outcomes were inconsistent between these two studies and original data were inaccessible, the results could not be pooled.

Three studies were excluded from the meta-analysis due to inconsistencies in the reported outcome measures. Two retrospective cohort studies [26, 27] used maximum efficacy (efficacy of > 3 months duration) and minimum side effects (side effects of < 2 weeks duration) as the evaluation criteria. Steven et al. [26] assessed the optimal effect and side effect duration in 45 patients receiving the same botulinum toxin dose. This was accomplished by comparing the number of injections resulting in an efficacy duration beyond 3 months (i.e., optimal effect) versus under 3 months, and duration of side effects under 2 weeks (i.e., optimal side effects) versus beyond 2 weeks, as well as the number of injections with or without optimal effect and side effects. The results suggested unilateral injection was more frequently associated with both optimal effect and side effects.

Recognizing that the small sample sizes and shortened timelines of the previous studies could not adequately reflect dynamic changes in injections arising from individual patient differences, Ishaan et al. [27] analyzed data from 4023 injections in 272 ADSD patients treated with botulinum toxin injections between 1994 and 2018. In contrast with the previous study [26], this longitudinal retrospective analysis found that bilateral injections were more frequently associated with optimal effect and side effects.

One non-randomized controlled study [24] employed an objective acoustic assessment of unilateral and bilateral treatment efficacy by comparing voice acoustic parameters at three time points (pre-injection, 1 week post injection, and 4 weeks post injection). They found that all voice acoustic parameters except signal-to-noise ratio improved 1 week after unilateral injection, whereas only voice break factor significantly improved after bilateral injection. At 4 weeks post injection, parameters in the unilateral group were closer to normal values compared to the bilateral group. Therefore, unilateral injection resulted in better and more rapid improvement in acoustic parameters.

The efficacy of botulinum toxin injections for ADSD must be weighed against side effects including breathiness, dysphagia, and coughing. Accordingly, most of the studies included in this report discussed efficacy in light of the side effect profile. After sensitivity analysis was used to exclude articles with the greatest heterogeneity, pooled results showed that compared to unilateral injections, bilateral botulinum toxin injections was associated with longer duration of breathiness and higher incidence of dysphagia. There was insufficient evidence for the reporting of cough and complete voice loss due to the small number of studies and low sample sizes.

Notably, although the results of this report can inform dynamic adjustment of injection protocols during the treatment cycle, they do not present a simple solution to the choice of unilateral versus bilateral approach for the initial injection, since the included studies did not concurrently correlate efficacy and side effects with injection approaches. Surveys indicate that 87% of U.S. physicians prefer bilateral injections for patients as the initial treatment [28], mainly due to patient demand. Ishaan [20] and Steven et al. [26] categorized treatments by the number of injections and correlated the optimal efficacy (> 3 months) with optimal side effects (< 2 weeks) to compare the number of unilateral versus bilateral injections that concurrently resulted in an optimal effect/side effect combination. This analysis informs the choice of initial injection approach; however, the results of these two studies were inconsistent, warranting further investigation into the analytical approach. Additionally, Zwirner et al. [24] evaluated aerodynamic parameters at three timepoints and found significantly higher mean air flow rate 1 week after bilateral compared to unilateral injections, which is consistent with the higher incidence of dysphagia observed following bilateral injections.

Our study has some limitations. First, the included studies contained patients with both ADSD and comorbid essential vocal tremor [18]. ADSD and essential tremor both manifest as impaired neuromuscular control of the larynx. Steven et al. [26] found no significant difference in the desirable profile of maximum efficacy with minimum side effects between unilateral versus bilateral injections in ADSD patients with essential tremor. Therefore, studies containing patients with both conditions were not excluded, although efficacy and side effects specifically for ADSD cannot be determined. Second, the included non-randomized controlled studies and cohort studies were found to have a moderate-to-high risk of bias based on quality and bias assessment. Third, there was high heterogeneity among the studies, including botulinum toxin type, injection doses, and treatment cycles, and the number of studies was small after sensitivity analysis. Additionally, subgroup analysis was not possible due to the small number of studies and lack of subgroup data.

The goal of this study was to investigate which injection approach results in better efficacy and fewer adverse effects for ADSD. Due to the currently limited and low-quality evidence, the results evaluated here can only provide a reference for dynamic adjustment of injection protocols during serial botulinum toxin treatment cycles. Suggestions for improving future research in this field are provided based on limitations of the included studies as follows:

First, a large degree of heterogeneity in injection doses existed across studies. Selection of initial doses were varied and not justified in most studies. During serial botulinum toxin treatments, earlier studies used fixed doses for comparison, while later studies dynamically adjusted doses based on physician experience and patient responses, introducing heterogeneity. We suggest reporting injected doses as low/medium/high dose groups to enable future subgroup meta-analyses by dose.

Second, there was a notable lack of ADSD-specific outcome measures. Efficacy assessments relied primarily on subjective measures such as duration of voice improvement and injection interval, with few studies incorporating objective acoustic analyses. Severity of voice impairment in particular was largely overlooked. Based on proposed core outcome measurement sets (patient-reported outcome measures, perceptual analysis, acoustic analyses, visual analyses and aerodynamic measurements) for common voice disorders by the European Laryngological Society (ELS) [29], core measurement tools for ADSD include: a PROM (the VHI) and perceptual rating measurements (voice breaks + the GRBAS: grade, roughness, breathiness, and strain, with less importance placed on asthenia). Acoustic measures were added as optional due to conflicting evidence. Future studies should combine subjective and objective assessments.

Third, there were numerous inconsistencies in study duration and design; follow-up periods ranged from 3 months for a single injection, to many years administering more than 20 injections. Lee et al. [19] noted that the difference in injection intervals between unilateral and bilateral groups became minimized over time, supporting the need proposed herein to analyze short-versus long-term studies. Given the predominance of retrospective cohort studies, which are prone to bias, short-term randomized controlled trails and prospective cohort studies with long-term follow-up should be considered, as well as nonrandomized self-controlled before-after studies using the cyclic nature of botulinum toxin injections.

Finally, there was a general lack of subgroup analysis in the injection approach, which is problematic as the unilateral/bilateral dichotomy is overly simplistic. Some studies used alternating unilateral injections [12, 18, 19] versus consistent unilateral injections, warranting comparison. Other variations, including dose equality for bilateral injections [18], should also be considered for future subgroup analyses using more granular groupings.

## Conclusion

Our meta-analysis and critical review of this field showed that bilateral botulinum toxin injections for ADSD showed a longer duration of vocal improvement, a longer breathy voice duration and a higher dysphagia occurrence and duration than unilateral injections. However, the insufficient evidence and significant heterogeneity among studies suggests that a variety of methodological improvements are required in this field, including increasing the number of studies and sample size, refining subgroup analyses (e.g., dose, approach, and duration), diversifying study design, and utilizing ADSD-specific outcome measures to her quality evidence.

## Data Availability

Not applicable.
